# Multi-fidelity graph neural networks for predicting toluene/water partition coefficients

**DOI:** 10.1186/s13321-025-01057-6

**Published:** 2025-08-08

**Authors:** Thomas Nevolianis, Jan G. Rittig, Alexander Mitsos, Kai Leonhard

**Affiliations:** 1 Institute of Technical Thermodynamics, RWTH Aachen University, 52062 Aachen, Germany; 2 Process Systems Engineering, RWTH Aachen University, 52074 Aachen, Germany; 3 Institute of Climate and Energy Systems ICE-1: Energy Systems Engineering, Forschungszentrum Jülich GmbH, 52425 Jülich, Germany; 4 JARA-SOFT, 52425 Jülich, Germany

**Keywords:** Graph neural network, Multi-fidelity learning, Partition coefficients

## Abstract

Accurate prediction of toluene/water partition coefficients of neutral species is crucial in drug discovery and separation processes; however, data-driven modeling of these coefficients remains challenging due to limited available experimental data. To address the limitation of available data, we apply multi-fidelity learning approaches leveraging a quantum chemical dataset (low fidelity) of approximately 9000 entries generated by COSMO-RS and an experimental dataset (high fidelity) of about 250 entries collected from the literature. We explore the *transfer learning*, *feature-augmented learning*, and *multi-target learning* approaches in combination with graph neural networks, validating them on two external datasets: one with molecules similar to training data (EXT-Zamora) and one with more challenging molecules (EXT-SAMPL9). Our results show that *multi-target learning* significantly improves predictive accuracy, achieving a root-mean-square error of 0.44 $\log {P}$ units for the EXT-Zamora, compared to a root-mean-square error of 0.63 $\log {P}$ units for single-task models. For the EXT-SAMPL9 dataset, *multi-target learning* achieves a root-mean-square error of 1.02 $\log {P}$ units, indicating reasonable performance even for more complex molecular structures. These findings highlight the potential of multi-fidelity learning approaches that leverage quantum chemical data to improve toluene/water partition coefficient predictions and address challenges posed by limited experimental data. We expect the applicability of the methods used beyond just toluene/water partition coefficients.

**Supplementary Information:**

The online version contains supplementary material available at 10.1186/s13321-025-01057-6.

## Scientific contribution

We investigate the benefits of transfer learning, feature-augmented learning, and multi-target learning approaches in combination with graph neural networks for the prediction of toluene–water partition coefficients. We show how a combination of a large number of cheap data from the semi-empirical COSMO-RS model with a few high-fidelity experimental data and multi-target learning efficiently leads to machine learning models with broad applicability and low uncertainties of 0.44 to 1.02 log units in the partition coefficient, depending on the test set.

**Supplementary Information:**

The online version contains supplementary material available at 10.1186/s13321-025-01057-6.

## Introduction

The partition coefficient $\log {P}$ of neutral species between water and an organic species is an important physical property, playing a significant role in various fields such as drug discovery [1–4] and separation processes [5, 6]. This property captures the ratio of concentrations of a chemical species in two immiscible solvents. For pharmaceutical applications, the partition coefficient of an Active Pharmaceutical Ingredient (API) indicates its hydrophobicity/hydrophilicity and is thus a critical indicator for its pharmacokinetics and physical properties of potential drug candidates [7, 8]. In separation processes, the partition coefficient between water and an organic solvent is key for determining the most effective methods for separating species impacting both the yield and purity [9–11]. While water/octanol partition coefficients of neutral species are widely available, data for water and other organic solvents, such as toluene/water are limited. Toluene/water partition coefficients offer better physiological relevance compared to water/octanol [12, 13]. Consequently, models that predict toluene/water partition coefficients for a wide spectrum of neutral species are highly desired.

Existing computational methods, such as the COnductor like Screening MOdel for Real Solvents (COSMO-RS) [14, 15], Group Contribution (GC), and Molecular Dynamics (MD) have been employed to predict toluene/water $\log {P}$ of neutral species [16–23]. Recently, the SAMPL9 blind challenge [24] allowed different groups to compare such predictive methods against a set of 16 drug-like molecules for predicting toluene/water $\log {P}$. We also participated in the challenge using the COSMO-RS method. Among 18 contributions, we ranked second with an Root-Mean-Square Error (RMSE) of 1.24 $\log {P}$ units [25]. The best-performing method in the SAMPL9 blind challenge [24] achieved an RMSE of 1.12 $\log {P}$ units [24]. COSMO-RS is a semi-empirical model, partially physics-based, allowing application to any system, though its performance varies depending on the specific system and property being studied [26–28]. Nevertheless, COSMO-RS shows good agreement with experimental $\log {P}$ values in our dataset that we performed in this study, supporting our choice to use it for generating low-fidelity training data. Machine Learning (ML) offers new possibilities for predicting toluene/water $\log {P}$ by utilizing experimental data. Recent advances in ML such as Graph Neural Networks (GNN) models and transformers enable end-to-end learning of molecular properties directly from the structure and have demonstrated success across various applications [29–37]. The general idea is to find a representation of molecules, e.g., in the form of descriptors, strings, or graphs, which can be mapped to properties of interest by applying regressions methods. For instance, in predicting the toluene/water partition coefficient of APIs as a post-SAMPL9 study, Zamora et al. [38] used a variety of molecular descriptors—related to the topological structure and properties such as the Ghose–Crippen water/octanol partition coefficient—on which they fitted a multiple linear regression model for the 251 experimental $\log {P}$ values from their collected dataset. These 251 experimental toluene/water $\log {P}$ of neutral species [38] are currently the largest available dataset in the literature. This multiple linear regression model achieved an RMSE of 1.05 $\log {P}$ units on the test dataset and an RMSE of 0.86 $\log {P}$ units on the SAMPL9 dataset [38]. These promising results are constrained by the limited amount of training data, which may restrict the model’s broader applicability and potentially its effectiveness across diverse solutes and $\log {P}$ ranges. The direct prediction of toluene/water $\log {P}$ of neutral species using ML therefore remains limited due to data scarcity, necessitating the exploration of alternative approaches.

To address scarcity of molecular property data, previous literature studies [32, 39–41] have employed various multi-fidelity learning approaches. A recent review by Qian et al. [42] summarizes the different multi-fidelity methods, suggesting that pretraining models on low fidelity data such as a large dataset derived from Quantum Chemical (QC) calculations and semi-empirical models, followed by fine-tuning with high fidelity data such as experimental data, can significantly enhance their applicability and reliability in predicting molecular properties. In particular, three multi-fidelity approaches are promising in molecular ML: *transfer learning*, *feature-augmented learning*, and *multi-target learning* [42]. *Transfer learning* leverages pretrained models to improve predictions, *feature-augmented learning* integrates predictions as additional features, and *multi-target learning* simultaneously predicts multiple related properties. To this end, to overcome the challenges posed by limited experimental data in predicting toluene/water $\log {P}$ of neutral species, we investigate these three multi-fidelity learning approaches that leverage QC and experimental data to increase the effectiveness of GNN models.

We apply various ML models and multi-fidelity learning approaches to predict the toluene/water $\log {P}$ of neutral species. Initially, we generate a low fidelity QC dataset consisting of approximately 9000 toluene/water $\log {P}$ values of neutral species using the COSMO-RS approach, which we chose due to its balance of accuracy and computational efficiency. We use this dataset to pretrain GNN models, so they encompass a wide range of chemical classes and atom types. We then fine-tune and test the pretrained GNN models with different multi-fidelity learning approaches using the high fidelity datasets of Zamora et al. [38] and the SAMPL9 blind challenge. Specifically, a part of the Zamora dataset, comprising 212 out of 250 experimental $\log {P}$ values, is used for fine-tuning while the remaining part 38 out of 250 is reserved for testing, similar to the approach taken with the SAMPL9 dataset, which includes 16 experimental $\log {P}$ values. The Zamora dataset originally contained 251 values, but we removed one duplicate molecule that appeared as both entry 79 (Aflukin) and entry 266 (Quinine) in External-SAMPL9 (EXT-SAMPL9) [38]. Next, we compare the GNN models with a GNN trained only on the experimental data and additional semi-empirical and data-driven approaches for the prediction of toluene/water $\log {P}$. Finally, we discuss the strengths and limitations of the different approaches. Thereby, we address how multi-fidelity strategies leveraging both QC and experimental values can play a crucial role in ML for accurately predicting molecular properties, especially when only a limited amount of experimental data is available.

## Dataset

We first present the low and high fidelity datasets of toluene/water partition coefficients and describe the data splitting process for training and testing of the computational methods. An overview of the datasets is shown in Table 1. The SMILES from all molecules used in this study are provided in the supporting information as a CSV file.

**Table 1:** Overview of $\log {P_{\mathrm {tol/w}}}$ datasets used for model (pre-)training and testing

Name	Number of data points	Origin
LF-QC^a^	8891	QC
HF-Exp [38]	212	Exp.
EXT-Zamora [38]	38	Exp.
EXT-SAMPL9 [24]	16	Exp.

^a^ The LF-QC set is generated in this work and is not publicly available due to licensing restrictions. We describe how to generate the LF-QC set in the text

### Low fidelity-quantum chemical dataset

To generate the Low fidelity-quantum chemical (LF-QC) dataset of $\log {P}$ values, we initially collect molecules represented by SMILES strings from the iBonD database [43], covering a diverse range of chemical classes and atom types. The iBonD database is chosen because it contains many drug-like molecules similar to those in the experimental datasets investigated in this work while also covering a broad spectrum of chemical diversity. The molecules are selected on the basis of standard ranges of acid dissociation constants. The final selection consists of molecules, predominantly featuring substituted benzoic and phenolic acids, alkyl carboxylic acids, alkylamines, and derivatives of pyridine and aniline. We then use these SMILES strings as input to obtain the 3D geometric structures using the software RDKit [44, 45]. Next, we optimize the molecular structures obtained from RDKit at the GFN2-xTB level of theory [46]. We further refine the geometries of each molecule in the COSMO state using COSMOconf 23 [47], with the BP86/TZVPD parametrization and FINE COSMO cavity [48–50]. Finally, we calculate the $\log {P_{\mathrm {tol/w}}}$ values for each molecule at 25 °C and at low finite dilution (0.0002 mol%) using COSMOtherm 23 [51], based on the difference in chemical potential between the water and toluene phases. We utilize small finite fractions of the molecules in both the aqueous phase and toluene to match the solute concentration range used in the experimental studies, which is 2.0–0.5 mM [52]. The error of $\log {P}$ in the LF-QC dataset is determined by propagating the uncertainties of the solvation free energies in water and toluene using Eq. 2. Given the uncertainty of 0.45 kcal mol$^{-1}$ [53] for the solvation free energy, the resulting error in $\log {P}$ is 0.47 $\log {P}$ units. The LF-QC dataset consists of 8891 molecules (see Table 1). The LF-QC set is not publicly available due to licensing restrictions. Consultation with the commercial software provider confirmed that data sharing is not permitted under our academic license terms. However, the $\log {P_{\mathrm {tol/w}}}$ values for each molecule in the LF-QC can be generated by applying the described approach to the provided SMILES strings, which are available in the supporting information as a CSV file.

### High fidelity-experimental dataset

The High fidelity-experimental (HF-Exp) dataset is obtained from Zamora et al. [38] who determined the partition coefficients $\log {P_{\mathrm {tol/w}}}$ through sample titrations, following a procedure similar to that used for aqueous acid dissociation constants determination but in the presence of varying amounts of the partitioning solvent. All measurements were conducted at 25 °C under an inert gas atmosphere, with at least three titrations performed for each compound to ensure accuracy. The solute concentration range estimations are based on the details provided in the experimental study [38, 52]. While these studies do not report the uncertainty of the toluene/water partition coefficient measurements, similar methods used for octanol/water partition coefficients typically report uncertainties around 0.04 $\log {P}$ units [54]. Therefore, it is reasonable to expect a similar level of uncertainty for the toluene/water measurements. An additional uncertainty arises from the fact that experimental concentrations are not provided for individual molecules, resulting in the calculations potentially being at slightly different concentrations. For most molecules, this difference will be negligible, but for molecules forming dimers in the toluene phase, the discrepancy can be in the order of 2 $\log {P}$ units [25]. The HF-Exp dataset consists of 212 molecules (see Table 1).

### External Zamora and SAMPL9 datasets

The External-Zamora (EXT-Zamora) and EXT-SAMPL9 datasets are taken from previous studies [24, 38]. The experiments conducted to measure the $\log {P_{\mathrm {tol/w}}}$ values in these datasets follow similar protocols to those used for obtaining the HF-Exp dataset. The EXT-Zamora and EXT-SAMPL9 datasets consist of 38 and 16 molecules, respectively (see Table 1).

### Dataset comparison and analysis

**Fig. 1:**
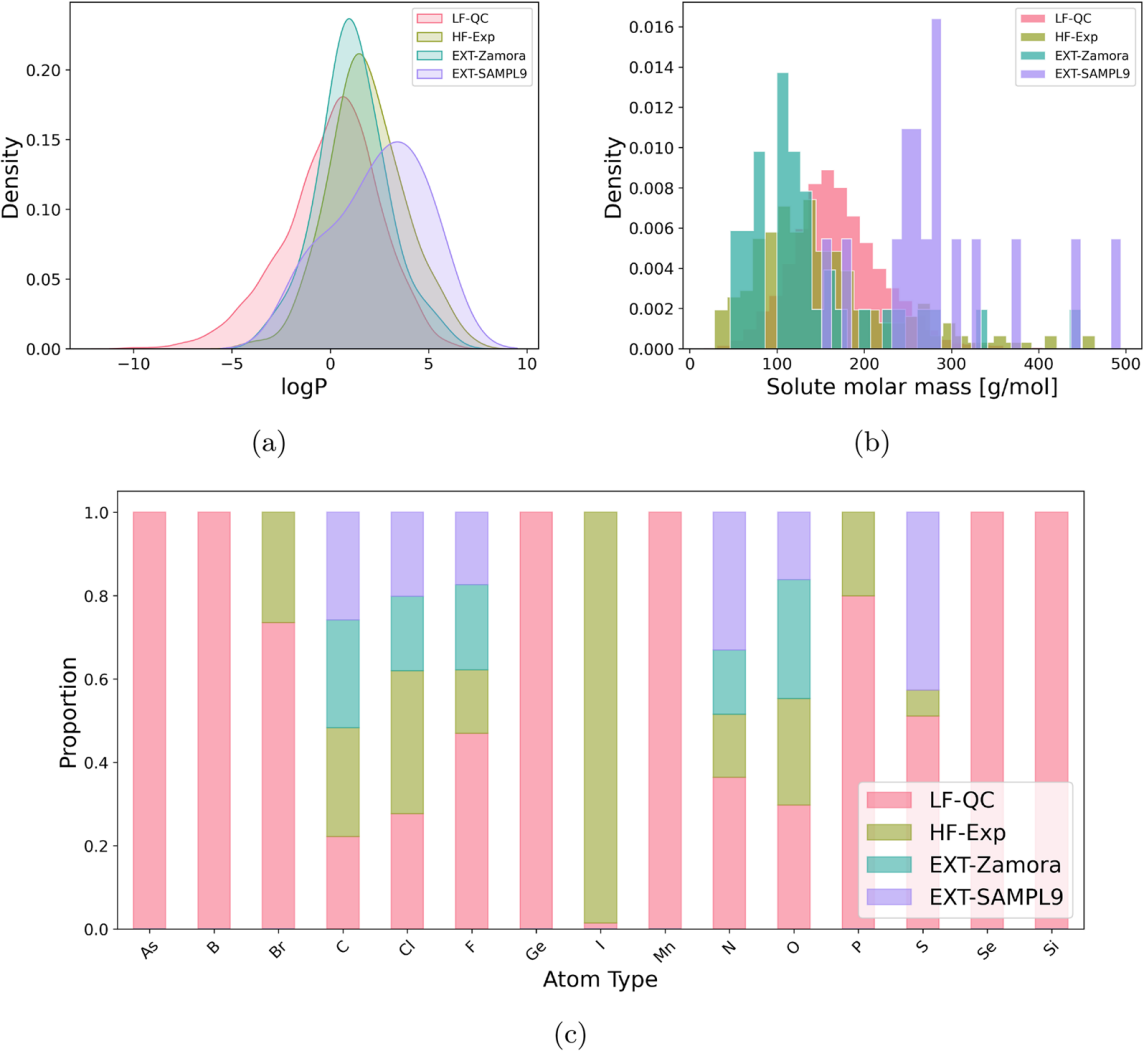
Comparison of chemical properties across four datasets:  LF-QC,  HF-Exp,  EXT-Zamora, and  EXT-SAMPL9. The subfigures show **a** density plots of $\log {P}$ values, **b** density distribution of molar masses, and **c** analysis of atom type distributions

Figure 1a shows the density distributions of $\log {P}$ values for the LF-QC, HF-Exp, EXT-Zamora, and EXT-SAMPL9 datasets. The LF-QC dataset (red) shows a wide distribution range from − 10 to 7, reflecting the extensive chemical diversity captured by the Quantum Mechanics (QM) dataset. The HF-Exp dataset (green) and EXT-Zamora dataset (cyan) have a more narrow and peaked distribution centered around − 1 to 3 $\log {P}$ values, indicating that the experimental measurements are focused on a more homogenous set of species. The EXT-SAMPL9 dataset (purple) peaks around − 1 to 3 $\log {P}$ values and 3 to 6 $\log {P}$ values, indicating differences in the molecules compared to the other datasets. The broad range of the LF-QC dataset shows the variability in computational predictions, while the narrower distributions of the experimental datasets (HF-Exp, EXT-Zamora, EXT-SAMPL9) reflect controlled conditions and specific chemical spaces. This variation is crucial for evaluating the performance and generalizability of predictive models across different types of data.

Figure 1b depicts the density distributions of solute molar masses for the LF-QC, HF-Exp, EXT-Zamora, and EXT-SAMPL9 datasets. The LF-QC dataset (red) shows a peak around 170 g mol$^{-1}$, indicating a relatively uniform distribution of molecules. The HF-Exp dataset (green) and the EXT-Zamora dataset (cyan) have a peak around 110 g mol$^{-1}$, suggesting a range of smaller molecule sizes. The EXT-SAMPL9 dataset (purple) displays a peak at higher molar masses, around 300 g mol$^{-1}$, indicating a tendency towards larger molecules.

Figure 1c shows the normalized distribution of different atom types across the LF-QC, HF-Exp, EXT-Zamora, and EXT-SAMPL9 datasets. This distribution is defined as the frequency of each atom type appearing in the datasets, adjusted so that the total frequency adds up to one. The LF-QC dataset (red) exhibits a broad distribution with significant representation across various atom types, highlighting its diverse chemical composition. The HF-Exp dataset (green) shows a more constrained distribution, indicating a focus on a narrower range of chemical species. The EXT-Zamora (cyan) and EXT-SAMPL9 (purple) datasets display even more distinct distributions, with the EXT-SAMPL9 dataset showing significant representation of specific atom types. This comparison highlights the diverse chemical compositions and focuses of the datasets, with LF-QC covering a wide array of atom types, while the experimental datasets (HF-Exp, EXT-Zamora, EXT-SAMPL9) are more specialized.

## Methodology

Next, we present the different computational methods, both semi-empirical and data-driven that we explore for predicting toluene/water partition coefficients. We choose COSMO-RS, a physics-based model, to generate low fidelity data because it performs better than the other available methods like GC and MD. Based on this low fidelity data, we develop several multi-fidelity ML approaches to address the issue of limited high fidelity experimental data.

### COSMO-RS

COSMO-RS is a computational model utilized for predicting thermodynamic properties and solvation behavior of molecules in solution. It combines quantum chemistry and statistical thermodynamics to estimate the chemical potentials of components in a system [14, 15]. Molecules are represented by surface segments, with segment interactions approximated as independent entities. The model relies on the $\sigma$-profile calculated from quantum chemical calculations, to predict the properties of interest. For a detailed description of COSMO-RS, we refer the interested reader to Refs. [55–59].

The logarithm of the toluene/water partition coefficient $\log {P}$ can be calculated according to

1 $$\begin{aligned} \log {P_{\mathrm {tol/w}}} = \log { \bigg ( \frac{\mathrm {[S]}_{\textrm{tol}}}{\mathrm {[S]}_{\textrm{w}}} \bigg ) } \,, \end{aligned}$$

where $\mathrm {[S]}_{\textrm{tol}}$ and $\mathrm {[S]}_{\textrm{wat}}$ are the concentrations of a solute [S] in toluene and water, respectively. In the COSMO-RS framework, the toluene/water $\log {P}$ is calculated according to [59, 60]

2 $$\begin{aligned} \log {P_{\mathrm {tol/w}}} = \frac{\mathrm {\Delta G}_{\textrm{Transfer}}}{R T \ln {10}} = \frac{ \mathrm {\Delta G}_{\textrm{w}}^{\textrm{solv}} - \mathrm {\Delta G}_{\textrm{tol}}^{\textrm{solv}} }{R T \ln {10}} \,, \end{aligned}$$

where $\mathrm {\Delta G}_{\textrm{Transfer}}$ is the transfer free energy of a solute from the pure aqueous phase to toluene. *R* is the gas constant and *T* is the temperature. $\mathrm {\Delta G}_{\textrm{w}}^{\textrm{solv}}$ and $\mathrm {\Delta G}_{\textrm{tol}}^{\textrm{solv}}$ are the solvation free energies of a solute in water and toluene, respectively. For all calculations, the temperature of 25 °C and the reference state of 1 mol L$^{-1}$ in the liquid and the gas is used.

Alternatively, the partition coefficient at infinite dilution can be calculated from infinite dilution activity coefficients $\gamma ^{\infty }$ and liquid molar volumes $\nu$ of toluene and water:

3 $$\begin{aligned} \log {P^{\infty }_{\mathrm {tol/w}}} = \log {\frac{\gamma _{\textrm{w}}^{\infty ,s}}{\gamma _{\textrm{tol}}^{\infty ,s}} \frac{\nu _{\textrm{w}}}{\nu _{\textrm{tol}}}} \end{aligned}$$

We also evaluated openCOSMO-RS [22, 23] as an open-source alternative but found it produced significant errors (see Table 3 and supporting information for more detail). This performance gap likely stems from openCOSMO-RS currently being limited to single conformers, which is particularly problematic for polar molecules that require multiple conformer consideration for accurate predictions. Development of conformer ensemble capabilities for openCOSMO-RS is currently underway to address this single-conformer limitation.

### Graph neural networks

GNN models learn properties directly from the molecular structure and have shown high prediction accuracies for a variety of both pure component [30, 61, 62] and mixture properties [32, 34, 63, 64]. Each molecule is represented as a graph with atoms as nodes and bonds as edges with corresponding feature vectors that contain atom and bond information, respectively. GNN models learn to extract local structural information about the molecular graph in graph convolutions that are then encoded into a vector representation. This molecular vector is then mapped to the property of interest by using a feedforward neural network. For a detailed description of GNN models, we refer the interested reader to overviews in Refs. [29, 36, 65, 66].

We use the Directed-Message Passing Neural Network (D-MPNN) model implemented in python library chemprop v1.7, which has achieved high accuracies in a variety of molecular property prediction tasks [67]. All datasets were split into 80-10-10 training-validation-test proportions using chemprop’s default random splitting. We use the default molecular features [67] and we tune the model hyperparameters of the chemprop library using 100 iterations of Bayesian optimization for hyperparameter search (see supporting information for more detail), with the test set remaining completely unseen during model development. The best set of parameters is chosen based on the validation error to train the final model, which is provided in the supporting information. We then explore different training approaches.

**Fig. 2:**
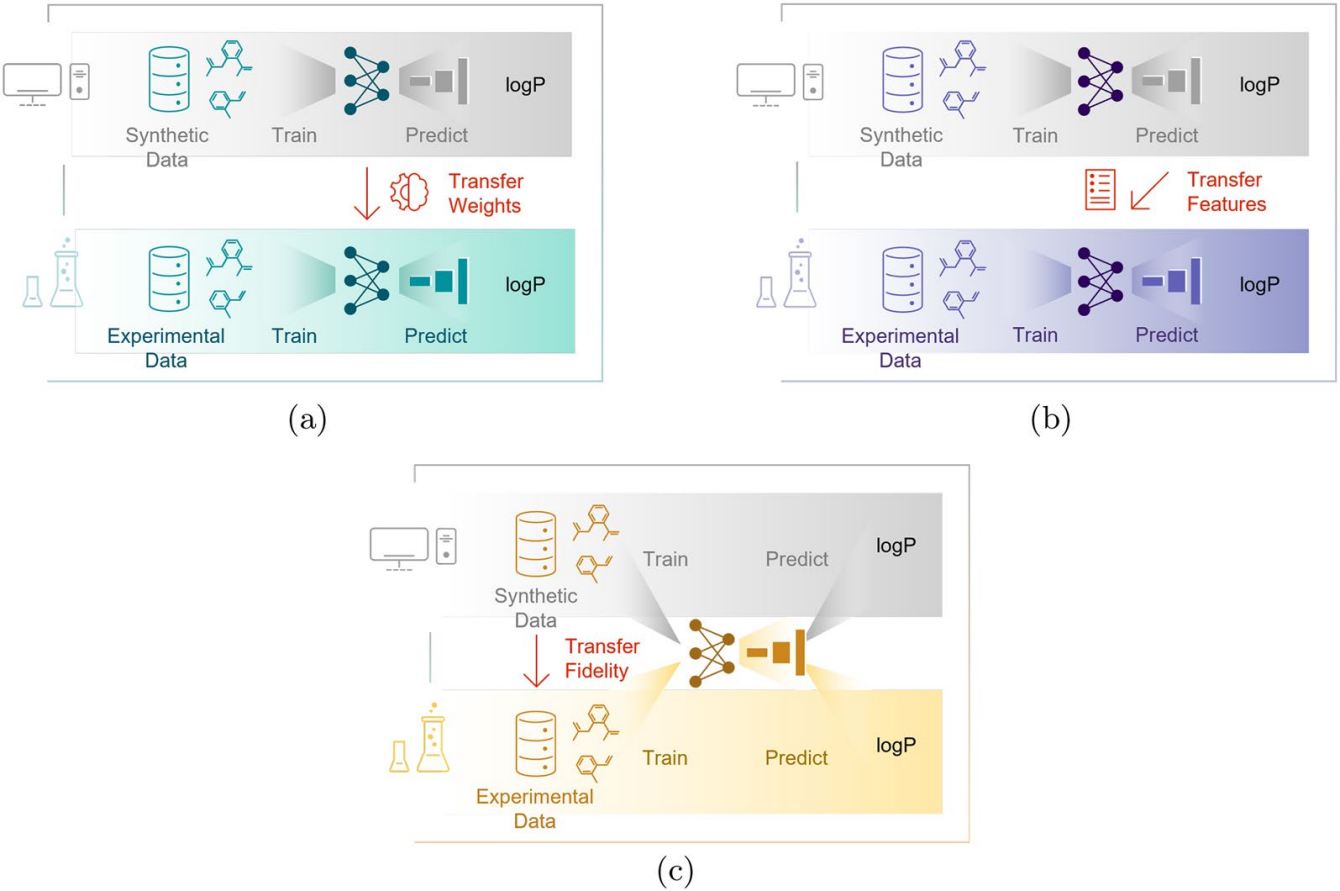
Overview of different multi-fidelity approaches for training the graph neural network models. Panels **a**–**c** depict the transfer learning, the feature-augmented learning, and the multi-target learning approach, respectively

We utilize three multi-fidelity approaches [42] to enhance the prediction of molecular properties: *transfer learning*, *feature-augmented learning*, and *multi-target learning* (see Fig. 2). *Transfer learning* (cf. Refs. [68, 69]) leverages pretrained models on LF-QC dataset to fine-tune predictions on the HF-Exp dataset. The idea is to use the low fidelity QC data (LF-QC) to develop a broadly applicable model and then employ the high fidelity experimental data (HF-Exp) to increase model’s accuracy, thus enhancing the model’s predictive capability with limited high fidelity data. *Feature-augmented learning* (cf. Ref. [41]) combines the HF-Exp dataset and LF-QC dataset: first a model is trained on the LF-QC dataset and then the predictions are used as an additional feature to existing ones for training a new model on the HF-Exp dataset. The purpose of *feature-augmented learning* is to integrate data of varying fidelities with high correlation to improve the predictive accuracy. *Multi-target learning* or multi-task learning (cf. Refs. [70, 71]) simultaneously predicts both experimental (HF-Exp dataset) and synthetic (LF-QC dataset) properties using a single model, aiming to exploit the interdependencies between different properties. This approach therefore aims to utilize information from multiple related tasks (predicted and experimental data) to improve the overall learning process and model robustness.

## Results & discussion

We now present a comparison of the D-MPNN prediction performance, focusing on the different multi-fidelity learning approaches, to conclude if one is more suitable than the others. We then compare these models with other existing models from the literature that can be used for toluene/water partition coefficient prediction to evaluate the multi-fidelity learning approaches overall.

### Comparison of multi-fidelity learning approaches

Table 2 first shows the performance of the D-MPNN models on the EXT-Zamora and EXT-SAMPL9 datasets. As described in Section “Dataset”, the EXT-Zamora contains molecules that are similar to the training sets (LF-QC and HF-Exp) in terms of molecular weight and $\log {P}$ range, thereby providing insight into the predictive capability within a similar molecular space. In contrast, the EXT-SAMPL9 dataset consists of relatively larger molecules, allowing us to evaluate the models’ generalization capabilities. We report the performance of various D-MPNN models, including single-task, *transfer learning*, *multi-target learning*, and *feature-augmented learning*.

**Table 2:** D-MPNN and Random Forrest (baseline) models performance comparison for EXT-Zamora [38] and EXT-SAMPL9 [24] datasets

Model	Mode	Dataset	Split	EXT-Zamora [38]		EXT-SAMPL9 [24]	
				RMSE	R$^2$	RMSE	R$^2$
D-MPNN (this work)	Single	HF-Exp	Random	0.63	0.86	1.32	0.65
D-MPNN (this work)	Single	LF-QC	Random	0.71	0.83	1.34	0.64
D-MPNN Transfer Learning (this work)	sequential	LF-QC + HF-Exp	Random	0.51	0.91	1.14	0.74
D-MPNN Multi-target (this work)	simultaneous	LF-QC + HF-Exp	Random	0.44	0.93	1.02	0.79
D-MPNN Feature-augmented (this work)	sequential	LF-QC + HF-Exp	Random	0.81	0.78	1.16	0.73
Random Forrest (baseline)	Single	LF-QC + HF-Exp	Random	0.88	0.74	2.51	− 0.26

The single-task D-MPNN model is trained on HF-Exp only and thus serves as a baseline to evaluate whether the inclusion of LF-QC data in the different multi-fidelity approaches can improve prediction accuracy. The single-task model achieves an RMSE of 0.63 $\log {P}$ units and R$^2$ of 0.86 on the EXT-Zamora and an RMSE of 1.32 $\log {P}$ units and R$^2$ of 0.65 on the EXT-SAMPL9 dataset. The lower accuracy observed on EXT-SAMPL9 dataset is expected, as this dataset tests the generalization to larger molecules. For completeness, we train also a single-task D-MPNN model on the LF-QC and the models shows comparable performance, with slight differences in RMSE and R$^2$ values (Table 2). For comparison with a traditional Quantitative StructureActivity Relationships (QSAR) method, we also trained a Random-Forest regressor on ECFP4 fingerprints generated from the combined LF-QC+HF-Exp set, retaining the experimental value whenever a molecule appeared in both sources. This fingerprint model attains an RMSE/$R^{2}$ of 0.88 $\log {P}$ units/0.74 on EXT-Zamora and 2.51 $\log {P}$ units/$-$0.26 on EXT-SAMPL9, markedly worse than any D-MPNN variant and thus underscoring the benefit of the graph-based approach.

Now considering the multi-fidelity approaches, we find that *transfer learning*, where the model is sequentially trained on the LF-QC dataset and HF-Exp dataset, shows an improvement over single-task training with an RMSE of 0.51 $\log {P}$ units and R$^2$ of 0.91 on the EXT-Zamora and an RMSE of 1.14 $\log {P}$ units and R$^2$ of 0.74 on the EXT-SAMPL9 dataset. The *multi-target learning* approach, which simultaneously trains on both LF-QC and HF-Exp datasets, performs even better, achieving an RMSE of 0.44 $\log {P}$ units and R$^2$ of 0.93 on the EXT-Zamora and an RMSE of 1.02 $\log {P}$ units and R$^2$ of 0.79 on the EXT-SAMPL9 dataset. The *feature-augmented learning* approach, which sequentially trains on LF-QC and HF-Exp datasets, does not perform as well as the *multi-target learning* approach, with an RMSE of 0.81 $\log {P}$ units and R$^2$ of 0.78 on the EXT-Zamora and an RMSE of 1.16 $\log {P}$ units and R$^2$ of 0.73 on the EXT-SAMPL9 dataset. It thus does not improve the predictive quality compared to the single-task model on the EXT-Zamora, but only on the EXT-SAMPL9 dataset. For the overall predictive quality in terms of RMSE and R$^2$, *multi-target learning* thus yields the highest improvement over single-task learning and is therefore most effective, see Table 2.

### Impact of molar mass

**Fig. 3:**
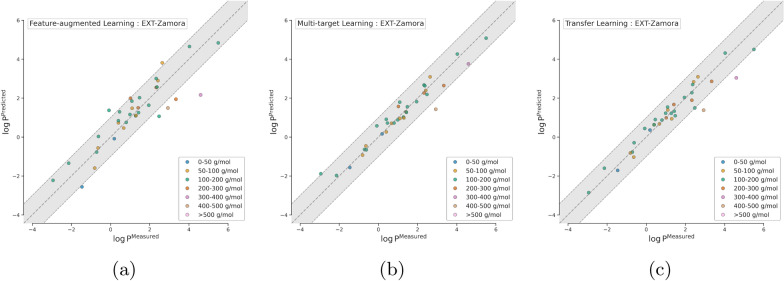
Comparison of the multi-fidelity learning approaches on EXT-Zamora dataset colored by molar mass range for **a** *feature-augmented learning*, **b** *multi-target learning*, and **c** *transfer learning*. Dashed lines indicate an error margin of ± 1 $\log {P}$ units

**Fig. 4:**
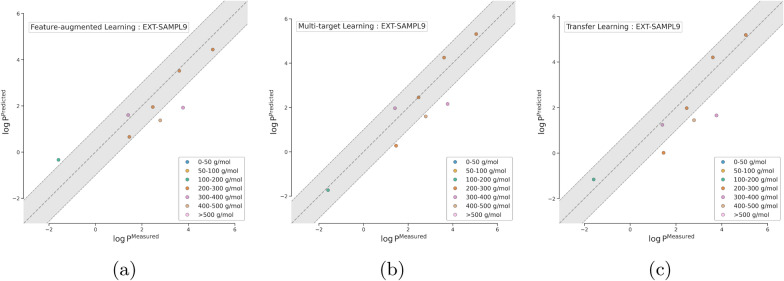
Comparison of the multi-fidelity learning approaches on EXT-SAMPL9 dataset colored by molar mass range for **a** *feature-augmented learning*, **b** *multi-target learning*, and **c** *transfer learning*. Dashed lines indicate an error margin of ± 1 $\log {P}$ units

Figures 3 and 4 further show the parity plots, i.e., predicted against the experimental data, of EXT-Zamora and EXT-SAMPL9 datasets for the different multi-fidelity approaches. The dashed lines indicate an error of ± 1 $\log {P}$ units. To analyze the impact of the molar mass on the performance of the models, we also indicate different weight ranges with colors.

For the EXT-Zamora dataset, the *multi-target learning* approach consistently shows the best performance across all molar masses. Only one molecule of 400 g mol$^{-1}$ to 500 g mol$^{-1}$ is out of the range of ± 1 $\log {P}$ units (see Fig. 3b). The *transfer learning* approach also performs well, though slightly less effectively for larger molecules > 300 g mol$^{-1}$. The *feature-augmented learning* approach, however, shows higher variability, particularly for the middle-weight range (100 g mol$^{-1}$ to 200 g mol$^{-1}$ and 200 g mol$^{-1}$ to 300 g mol$^{-1}$).

Similarly, for the EXT-SAMPL9 dataset, the *multi-target learning* approach maintains the best performance across most weight categories (see Fig. 4). It shows particularly strong results for light molecules and less strong results for heavier molecules. *Transfer learning* remains competitive but again shows slight performance degradation for heavier molecules. The *feature-augmented learning* approach continues to exhibit higher variability, especially for molecules in the 200 g mol$^{-1}$ to 300 g mol$^{-1}$ and > 500 g mol$^{-1}$.

Overall, the *multi-target learning* approach shows the highest predictive robustness across different molar masses.

### Impact of chemical classes

**Fig. 5:**
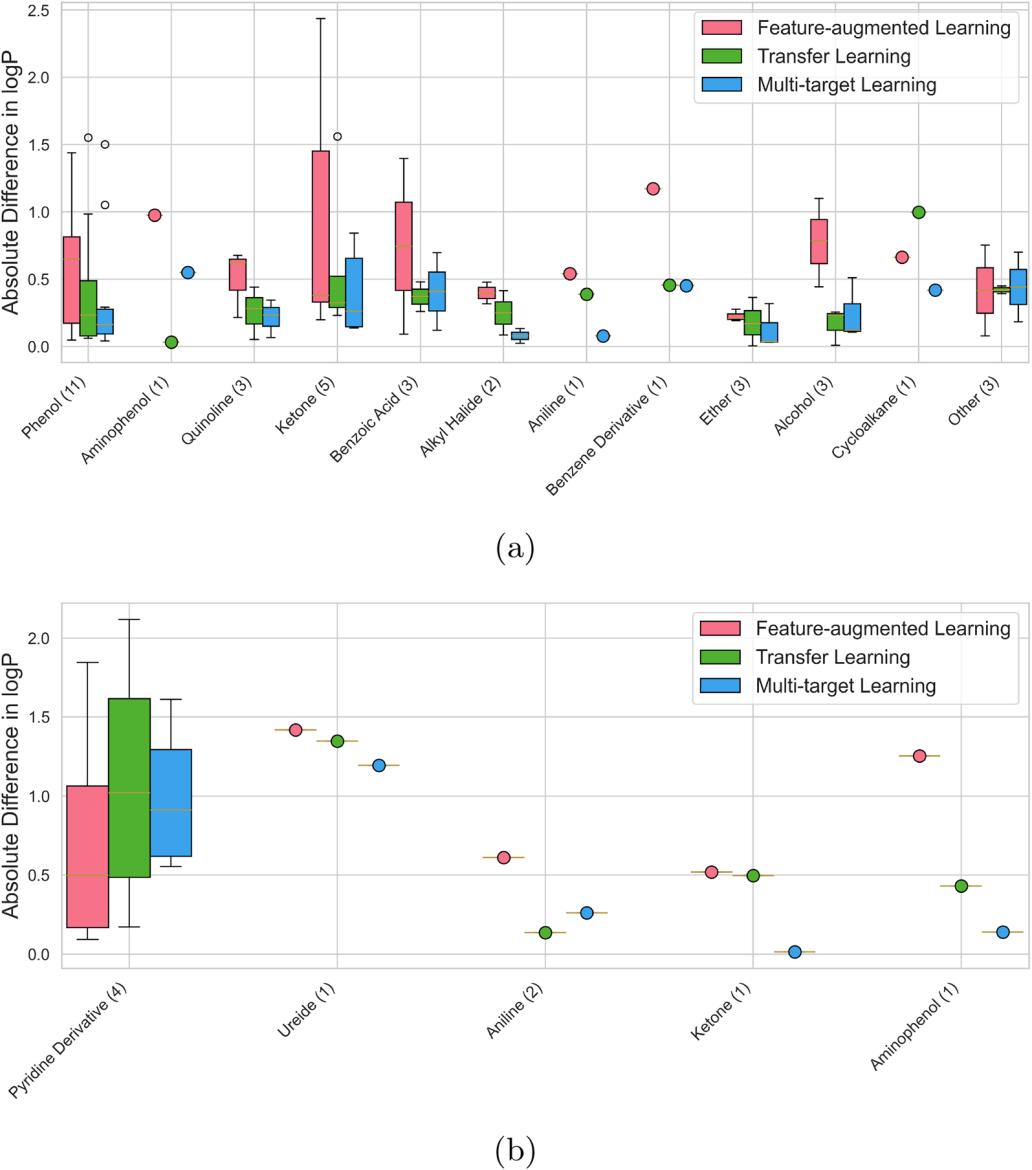
Predictive performance of the different multi-fidelity learning approaches across various chemical classes in the **a** EXT-Zamora and **b** EXT-SAMPL9 datasets. Numbers in parentheses indicate the number of molecules in each chemical class

We also investigate the model performance across different chemical classes, and illustrate the results in Fig. 5. To analyze the impact of chemical classes on model performance, we categorize molecules based on their chemical structures using SMARTS patterns and substructure matching. It is important to note that the overall number of molecules per class is very low (sometimes as few as one), indicating that additional data and further evaluations will be needed to confirm these findings. In Fig. 5, the boxes represent the interquartile range with lines indicating the median values and the whiskers extend to 1.5 times the interquartile range. The EXT-Zamora dataset features a diverse set of chemical classes, including 11 phenols, 5 ketones, 3 quinoline, 3 ethers, 3 alcohols, 2 benzoic acids, 2 alkyl halides, and one each of aminophenol, aniline, benzene derivative, and cycloalkane (5 molecules classified as other). The EXT-SAMPL9 dataset, in contrast, is less diverse compared to EXT-Zamora dataset. It is a smaller dataset comprising a limited range of chemical classes, containing 4 pyridine derivatives, 2 benzene derivatives, 2 anilines, and one each of phenol, ureide, ketone, aminophenol, and sulfonamide (3 molecules classified as other). An overview of the chemical class distributions in the LF-QC and HF-Exp datasets can be found in the supporting information.

The *multi-target learning* approach demonstrates the most consistent and lowest absolute differences in $\log {P}$ predictions across various chemical classes. For example, in the classes of alcohols, ethers, and alkyl halides, it shows significantly lower errors compared to *feature-augmented learning* and *transfer learning* approaches. Interestingly, *multi-target learning* shows a great agreement between predictions and experiments with a mean absolute lower lower than 0.5 $\log {P}$ units for the chemical classes aniline, ketone, and aminophenol for the EXT-SAMPL9 dataset and keeps the same consistency for the EXT-Zamora dataset except for the chemical class aminophenol. This indicates *multi-target learning* effectively captures the distinct characteristics of different chemical structures by leveraging both LF-QC and HF-Exp datasets during training.

*Transfer learning* also performs well across various chemical classes but shows higher variability in classes such as benzene derivatives and amides. This variability suggests that while *transfer learning* can improve model accuracy by integrating different data types, it may still face challenges in fully capturing the intricate properties of more complex molecules. For instance, the errors are more pronounced in the benzene derivatives class in the EXT-Zamora dataset, indicating a potential limitation in handling aromatic systems. This might be due to the fact that not enough data are available for the fine-tuning step, as Vermeire and Green [32] have shown that *transfer learning* can achieve a great agreement between predictions and experiments if enough high fidelity data are available.

The *feature-augmented learning* approach shows the highest absolute differences in several chemical classes, including ketones and benzene derivatives. This performance suggests that the method’s sequential training on LF-QC and HF-Exp datasets may not be as effective in capturing the detailed chemical properties required for accurate $\log {P}$ predictions. The higher errors in the ketone class, particularly in the SAMPL9 dataset, highlight the approach’s difficulty in balancing data contributions from different fidelities, especially for complex chemical structures. This indicates that *feature-augmented learning* requires careful handling to avoid poor performance in chemically diverse datasets, especially when few data is available for fine-tuning.

### Comparison to other models

We further compare the best performing D-MPNN model, *multi-target learning*, to other semi-empirical and data-driven models from the literature, as shown in Table 3. Specifically, we consider two GNN models that provide infinite dilution activity coefficient (AC) predictions, namely Gibbs-Duhem-informed (GDI)-GNNs trained on COSMO-RS activity coefficient data from our previous work [72] and the Gibbs–Helmholtz (GH)-GNN [73] trained on experimental infinite dilution activity coefficient (IDAC) data from the DECHEMA Chemistry Data Series [78]. To predict the partition coefficients, we employ the already trained models from Refs. [72, 73], using Eq. 3. We calculate the molar volumes with densities and molecular weights for toluene and water from the National Institute of Standards and Technology (NIST) Chemistry webbook [79]. We further include two GNN models based on the D-MPNN architecture trained on diverse datasets of COSMO-RS and experimental solvation Gibbs free energies, namely Solvation GNN [32] and DirectML [74]. Here, the partition coefficients are calculated using the already trained models from Refs. [32, 74] along with Eq. 2. All GNN models use an ensemble approach, i.e., the prediction of multiple models trained on different data splits are averaged to obtain a final prediction. In addition, we consider the MLR and RFR from Zamora et al. [38] that were fitted on the HF-Exp set. The partition coefficient values are taken directly from the original publication [38]. These two regression models use 11 input descriptors, including AlogP (octanol/water partition coefficient using Ghose–Crippen atomic contributions [80]), which shows a 58% correlation to the toluene-water partition coefficient, cf. [38]. Lastly, we compare to two semi-empirical models: COSMO-RS and MM/PBSA [24]. In the COSMO-RS approach [25], the geometry of each molecule is optimized at GFN2-xTB [46] level and further in the COSMO state using COSMOconf [81]. Next, the solvation free energies of the molecules are calculated in water and toluene at infinite dilution using COSMOtherm [82]. In the MM/PBSA approach, each molecule is optimized using QM, followed by molecular dynamics geometry optimization, and solvation free energies in water and toluene are calculated. In this case, the partition coefficient values are obtained directly from the original publication [24].

**Table 3:** Model performance comparison for EXT-Zamora [38] and EXT-SAMPL9 [24] datasets

Model	Mode	Dataset	Split	EXT-Zamora [38]		EXT-SAMPL9 [24]	
				RMSE	R$^2$	RMSE	R$^2$
D-MPNN Multi-target (this work)	Simultaneous	LF-QC + HF-Exp	Random	0.44	0.93	1.02	0.79
GDI-GNN^a^ by Rittig et al. [72]	Ensemble	COSMO-AC	–	**0.77**	**0.80**	1.56	0.51
GH-GNN^a^ by Sanchez Medina et al. [73]	Ensemble	DECHEMA IDAC	–	*1.23*	*0.48*	*1.69*	*0.43*
Solvation GNN^a^ by Vermeire and Green [32]	Ensemble	COSMO & exp. G	–	**0.27**	**0.97**	**1.07**	**0.77**
DirectML^a^ by Chung et al. [74]	Ensemble	COSMO & exp. G	–	**0.37**	**0.95**	**1.04**	**0.78**
MLR by Zamora et al.[38]	Single	exp	–	1.05	–	**0.86**	**0.85**
RFR by Zamora et al. [38]	Single	exp	–	1.13	–	**0.84**	**0.86**
COSMO-RS^a^ results from Nevolianis et al. [25]	–	COSMO	–	0.60	0.88	1.23	0.70
openCOSMO-RS^a^ by Müller et al. [23]	–	–	–	1.74	− 0.73	2.37	− 0.35
MM/PBSA^a^ by Amezcua et al. [24]	–	–	–	–	–	1.12	0.75
HANNA^a^ (Clapeyron.jl) by Walker et al. [75]	–	–	–	1.71	0.01	2.01	0.19
UNIFAC^a^ (thermo) by Bell et al. [76, 77]	–	–	–	3.34	− 2.80	3.89	− 2.01

^a^Models are not trained on partition coefficients

Some molecules of the test set are included in the training set (in bold)

*Some molecules of the test set might be included in the training set (the training set is not publicly available)*

The GDI-GNN model shows strong performance on EXT-Zamora dataset; however, its prediction accuracy is likely overestimated due to 16 of the 38 test set molecules being included in the training. In contrast, its performance on the EXT-SAMPL9 set, which has no overlap with the training data, is lower. The GH-GNN model generally shows lower performance, and since its training data is not publicly available, we could not identify potential overlaps of training and test data. Interestingly, activity coefficient GNN models are performing at level comparable to the top five models from the SAMPL9 challenge [24]. Yet, the activity coefficient GNN models show lower accuracy than the D-MPNNs directly trained on partition coefficients.

The Solvation GNN and DirectML models show high predictive quality; however, their accuracy is likely overestimated due to significant overlap between training and test molecules. For example, the experimental training data of Solvation GNN and DirectML contain, respectively, 29 (34 for pretraining) and 35 of the 38 molecules of EXT-Zamora, and, respectively, 4 (7 for pretraining) and 14 of the 16 molecules of EXT-SAMPL9. In fact, we observe a similar accuracy of the Solvation GNN and DirectML on EXT-SAMPL9 compared to the multi-target D-MPNN, although some molecules are already included in training, thus indicating at most comparable generalization capabilities.

The MLR and RFR models from Zamora et al. [38] show varying performance. Both models achieve higher accuracy on the EXT-SAMPL9 dataset compared to the EXT-Zamora dataset. The high predictive accuracy on the EXT-SAMPL9 indicates the effectiveness of using molecular descriptors when available training data is limited, which has also been reported in recent comparisons of ML/GNN models with and without using QC descriptors [83]. However, these models are typically limited in their generalizability to molecules dissimilar from the training data. The higher accuracy on the presumably more distinct EXT-SAMPL9 set compared to EXT-Zamora (cf. Section “Dataset”) is thus unexpected. In fact, we find that the experimental data used for fitting contains a duplicate entry with EXT-SAMPL9, indexed as entries 79 (Aflukin) and 266 (Quinine) [38]. This duplication might explain the better performance observed on the EXT-SAMPL9 dataset compared to the EXT-Zamora dataset. However, after retraining the models without the duplicate entry, the RMSEs for EXT-Zamora are 1.12 (MLR) and 1.04 (RFR), while for EXT-SAMPL9, they are 0.94 (MLR) and 0.90 (RFR), indicating that the duplication had only a minor impact on the results. We thus find lower accuracy of the MLR and RFR compared to the ML models for EXT-Zamora and slightly reduced accuracy for EXT-SAMPL9.

The COSMO-RS and MM/PBSA models from the SAMPL9 challenge show moderate performance on the EXT-SAMPL9 dataset but perform better on the EXT-Zamora dataset. Despite their performance, they are outperformed by the D-MPNNs with multi-fidelity learning. It is important to note that the SAMPL9 challenge reports different r$^2$ values, which are not coefficients of determination R$^2$; therefore, the R$^2$ values here have been recalculated for consistency. Additionally, we evaluated openCOSMO-RS as an open-source alternative to COSMO-RS, which showed limited accuracy on both datasets, primarily due to its current single-conformer limitation as discussed in the methods section (see supporting information). Last, the thermodynamic models HANNA and UNIFAC show limited performance on both datasets, with particularly poor R$^2$ values, suggesting that these methods may struggle with the molecular diversity present in these datasets. The absence of conformational flexibility in these approaches could be a significant limitation for larger, more flexible molecules where conformational effects play a crucial role in partition behavior.

## Conclusion

In this work, we investigated multi-fidelity learning approaches with GNN models for predicting toluene/water partition coefficients for which experimental data are only readily available in the order of a few hundred values. First, we used COSMO-RS to create a low fidelity dataset of partition coefficients for about 9000 molecules. The low fidelity data in combination with the available high fidelity experimental data was then utilized for training GNN models. Our results showed that *multi-target learning*, i.e., predicting low fidelity and high fidelity target properties with one GNN model, yields substantial accuracy increases to training a GNN model on the experimental data only and is superior to *transfer learning* and *feature-augmented learning*. We further found competitive accuracy of the multi-target GNN model compared to other predictive models, e.g., based on activity coefficients and solvation free energies, and other methods such as COSMO-RS. Overall, the comparison of the different approaches for partition coefficient predictions shows that direct training on $\log {P}$ data is most effective. Here, multi-fidelity learning in the form of *multi-target learning* substantially increases the predictive accuracy. This is particularly interesting as the *multi-target learning* approach presumably requires the least training and model changes, i.e., just an additional model output, and is thus straightforward to implement. Generating additional molecular property data through QC calculations for training predictive ML models like GNN models is thus highly promising to enhance the predictive quality when available experimental data is limited, such as for toluene/water partition coefficients. However, it is important to acknowledge that the availability of high fidelity data remains a significant challenge and the extrapolation to new chemical classes cannot be fully resolved with multi-fidelity learning approaches leveraging large low-fidelity datasets.

Future work could consider *multi-target learning* with low and high fidelity datasets for multiple molecular properties, e.g., combining activity coefficients, solvation free energies, and partition coefficients. For this, also thermodynamics relationships between the properties could be integrated into the model training and architecture, as, e.g., in [84, 85], aiming at more general predictive models.

## Supplementary Information

Supplementary Material 1.

## Data Availability

The datasets supporting the conclusions of this article are available in the Zenodo repository under DOI: 10.5281/zenodo.13236218. The SMILES for all molecules used in this study are provided in the supporting information as a CSV file, except for the LF-QC dataset, which is not publicly available due to licensing restrictions. The trained models are also not publicly available for the same reason. However, a Python notebook containing all the scripts and code to reproduce the results of this work is provided.
