# Supplementary material for: Multi-fidelity graph neural networks for predicting toluene/water partition coefficients
Source: J Cheminform. 2025 Aug 8;17:123. doi: 10.1186/s13321-025-01057-6 (PMC12333204; doi:10.1186/s13321-025-01057-6)
Supplement: Supplementary file 1 — Supplementary Material 1. [file 13321_2025_1057_MOESM1_ESM.pdf]

Supporting Information: Multi-fidelity graph neural networks for predicting toluene/water partition coefficients

Thomas Nevolianis,[†] Jan G. Rittig,[‡] Alexander Mitsos,[‡,¶,§] and Kai Leonhard[*,†]

†*Institute of Technical Thermodynamics, RWTH Aachen University, 52062 Aachen, Germany*

‡*Chair of Process Systems Engineering, RWTH Aachen University, 52074 Aachen, Germany*

¶*Forschungszentrum Jülich GmbH, Institute of Climate and Energy Systems ICE-1: Energy Systems Engineering, Jülich 52425, Germany*

§*JARA-SOFT, Aachen, Germany*

E-mail: Kai.Leonhard@ltt.rwth-aachen.de

Chemical class distribution in the LF-QC and HF-Exp datasets

Table S1: Overview of the chemical classes in the LF-QC dataset.

**Chemical Class**	**Count**
Ketone	2165
Other	1308
Phenol	1006
Pyridine Derivative	788
Aniline	744
Benzene Derivative	474
Benzoic Acid	399
Alcohol	345
Quinoline	285
Alkyl Halide	272
Sulfonamide	246
Pyrimidine Derivative	201
Aminophenol	175
Phenylbutylamine	102
Ether	93
Thiophene Derivative	92
Ureide	84
Phenylethanolamine	43
Cycloalkane	42
Indole	15
Piperazine Derivative	12

Table S2: Overview of the chemical classes in the HF-Exp dataset.

**Chemical Class**	**Count**
Phenol	47
Other	37
Ketone	29
Alkyl Halide	14
Benzene Derivative	13
Quinoline	11
Alcohol	10
Ether	10
Aniline	9
Phenylbutylamine	8
Pyridine Derivative	7
Benzoic Acid	7
Ureide	2
Aminophenol	2
Pyrimidine Derivative	2
Phenylethanolamine	2
Cycloalkane	2
Sulfonamide	1

Hyperparameters

Table S3: Searchable hyperparameters using `chemprop_hyperopt` taken from.[S1]

Keyword	Description
activation	The activation function used after each linear layer, when necessary.
aggregation	The aggregation function used when constructing a molecule-level representation from node-level representations.
aggregation_norm	The normalization factor if using norm aggregation.
batch_size	The minibatch size.
depth	The number of message-passing iterations.
dropout	The dropout probability after each layer in both the D-MPNN encoder and FFN.
ffn_hidden_size	The size of each hidden layer in the FFN.
ffn_num_layers	The number of layers in the FFN.
hidden_size	The message size in the D-MPNN encoder.
max_lr	The maximum learning rate used in the learning rate scheduler.
init_lr	The initial learning rate expressed as the ratio of init_lr to max_lr.
final_lr	The final learning rate expressed as the ratio of final_lr to max_lr.
warmup_epochs	The number of epochs over which to ramp up the learning rate from init_lr to max_lr, expressed as a fraction of the total training epochs.

Hyperparameter results

Table S4: Hyperparameter results for the *multi-target learning* method.

**Hyperparameter**	**Value**
activation	ReLU
aggregation	sum
aggregation_norm	76.0
batch_size	140
depth	4
dropout	0.05
ffn_hidden_size	300
ffn_num_layers	3
final_lr	7.88154413271554e-05
hidden_size	800
init_lr	6.193713383888547e-06
max_lr	0.004155920461904726
warmup_epochs	15

Table S5: Hyperparameter results for the *transfer learning* method.

**Hyperparameter**	**Value**
activation	ReLU
aggregation	norm
aggregation_norm	192.0
batch_size	50
depth	6
dropout	0.2
ffn_hidden_size	700
ffn_num_layers	3
final_lr	0.0026602019364376866
hidden_size	900
init_lr	5.3010992525841e-05
max_lr	0.004540534678184651
warmup_epochs	5

Table S6: Hyperparameter results for the *feature-augmented learning* method.

**Hyperparameter**	**Value**
activation	LeakyReLU
aggregation	mean
aggregation_norm	99.0
batch_size	30
depth	3
dropout	0.1
ffn_hidden_size	2400
ffn_num_layers	1
final_lr	0.00022766782311393923
hidden_size	2300
init_lr	1.145260591279654e-06
max_lr	0.00354533150456784
warmup_epochs	10

openCOSMO-RS

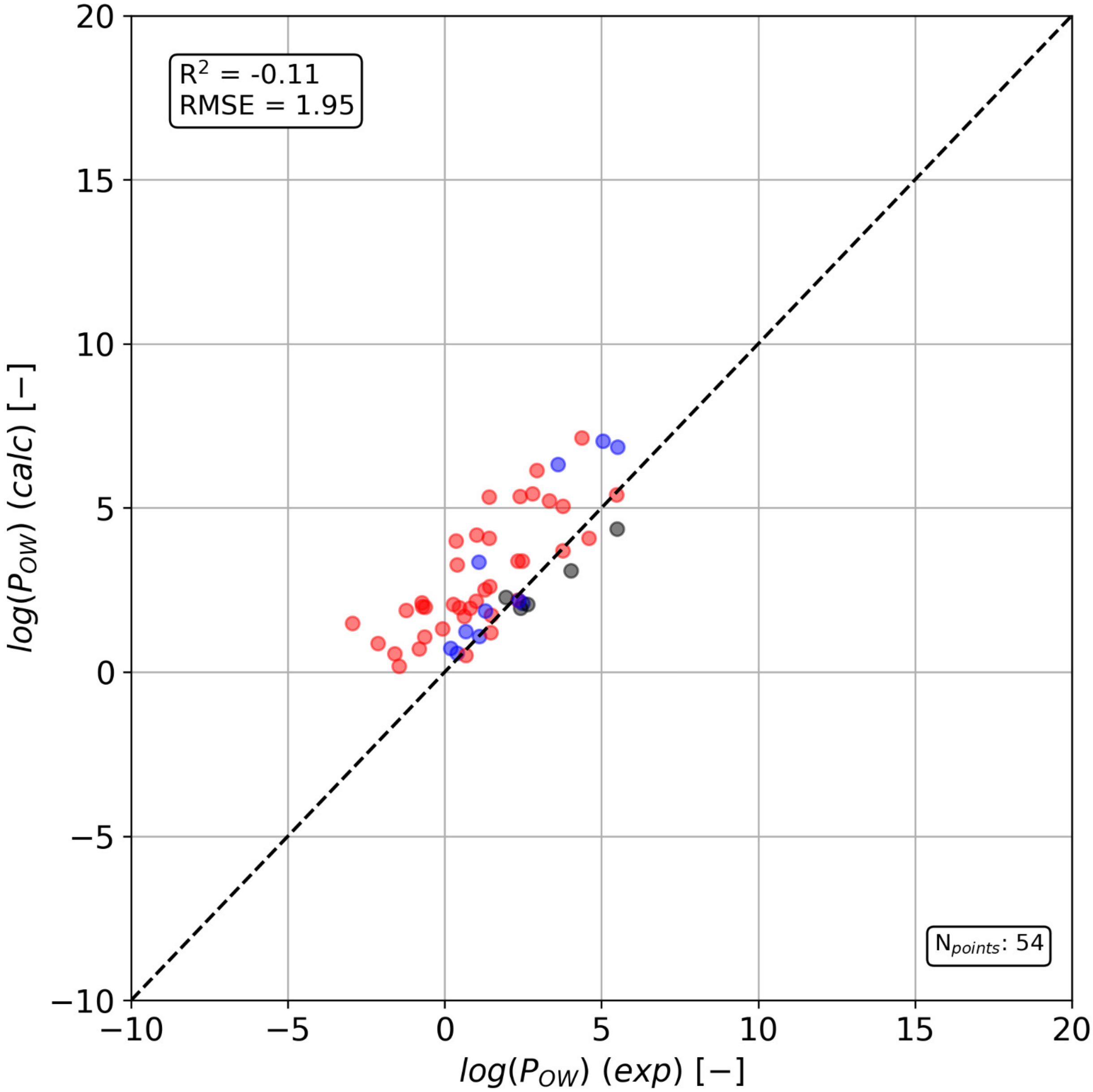

Figure S1: Parity plot for toluene/water partition coefficient from the EXT-Zamora and EXT-SAMPL9 datasets calculated with openCOSMO-RS 24a. Colors represent different solute types: (●) non-hydrogen-bonding, (●) hydrogen-bond acceptors, (●) hydrogen-bond donors, and (●) hydrogen-bond donors/acceptors.

References

(S1) Heid, E.; Greenman, K. P.; Chung, Y.; Li, S.-C.; Graff, D. E.; Vermeire, F. H.; Wu, H.; Green, W. H.; McGill, C. J. Chemprop: A machine learning package for chemical property prediction. *Journal of Chemical Information and Modeling* **2023**, *64*, 9–17.
